# Roles of Chloroplast Retrograde Signals and Ion Transport in Plant Drought Tolerance

**DOI:** 10.3390/ijms19040963

**Published:** 2018-03-23

**Authors:** Chenchen Zhao, Anthony M. Haigh, Paul Holford, Zhong-Hua Chen

**Affiliations:** 1 School of Science and Health, Western Sydney University, Penrith, NSW 2751, Australia; Chenchen.Zhao@westernsydney.edu.au (C.Z.); a.haigh@westernsydney.edu.au (A.M.H.); P.Holford@westernsydney.edu.au (P.H.); 2 Hawkesbury Institute for the Environment, Western Sydney University, Penrith, NSW 2751, Australia

**Keywords:** retrograde signalling, ion channels, pumps, cotransporters, stress responsive genes

## Abstract

Worldwide, drought affects crop yields; therefore, understanding plants’ strategies to adapt to drought is critical. Chloroplasts are key regulators of plant responses, and signals from chloroplasts also regulate nuclear gene expression during drought. However, the interactions between chloroplast-initiated retrograde signals and ion channels under stress are still not clear. In this review, we summarise the retrograde signals that participate in regulating plant stress tolerance. We compare chloroplastic transporters that modulate retrograde signalling through retrograde biosynthesis or as critical components in retrograde signalling. We also discuss the roles of important plasma membrane and tonoplast ion transporters that are involved in regulating stomatal movement. We propose how retrograde signals interact with ion transporters under stress.

## 1. Introduction

The world will need to feed more than 9 billion people by 2050 [1], thus food production needs to increase by at least 70% [2]. However, because of the decrease of land available for agriculture and of climate change, this will be a substantial challenge [3]. To meet this challenge, a better understanding of crop agronomy and physiology is needed together with the development of new germplasm allowing sustainable crop production under adverse environmental conditions such as drought. About half of earth’s land area is susceptible to drought [4], which is regarded as a sunstantial threat to global food security [5]. During evolution, plants have adopted many mechanisms to counteract drought. One significant mechanism is stomatal regulation, and highly responsive stomata have been suggested as key factors for the success of grasses in adverse environments [6]. During drought plants close their stomata, thereby reducing water uptake, which affects their normal physiological functioning and nutrient uptake from the soil and reduces their growth and yield [7]. Plants protect themselves in the short term by closing stomata [8] and in the long term by increasing the root/shoot ratio [9], root hydraulic conductance [10], thickness of leaf cuticle [11], stomatal development [12], and cuticular wax [13]. If these drought avoidance mechanisms are not successful, mechanisms to tolerate dehydration may be switched on. These mechanisms include ways of maintaining cell water content through ion accumulation [8], cell wall stiffening [14], production of protective compounds [15], metabolic changes, and detoxification of reactive oxygen species (ROS) [16]. However, to respond to drought, a plant must first perceive the stress and then transduce the related recognition events via signalling networks. As a result, the transcription of specific drought stress response genes occurs, leading to changes in physiological processes and systemically transducing further signals throughout the plant.

Apart from the dominant function of photosynthesis, chloroplasts also function as sensors of environmental stimuli such as drought stress and initiate signals that induce nuclear gene expression (NGE) across a range of evolutionarily important plant species [16]. The endosymbiotic theory suggests that a plant cell is the result of hundreds of millions of years of co-evolution of cyanobacteria and early eukaryotic cells, i.e., the host [17]. From this stable endosymbiotic relationship, cyanobacteria evolved into modern chloroplasts, and large numbers of cyanobacterial genes were transferred to the host nucleus during evolution [18]. However, around 100 genes involved in photosynthesis have been retained in chloroplasts [19]. Therefore, the expression of nuclear and chloroplast genes must be combined and coordinated for the cells to function efficiently [20]. Nucleus to plastid signalling is termed anterograde signals [21], and plastid-derived signals that target the regulation of NGE are called retrograde signals [22]. In addition to coordinating chloroplast functioning, there is evidence that retrograde signalling has a role in adaptation to stress. The progenitors of plastids would have contained functions helping plants respond to the environment through the expression of stress response genes [23].

Stomata evolved from early land plant species like mosses, function in regulating water potential and CO_2_ fixation and regulate drought tolerance [24]. A chloroplast-initiated retrograde signalling pathway has been identified as having a significant role in regulating stomatal movement, which greatly affects plant drought tolerance [25,26]. Plants have a complex signalling system to control stomatal opening that is driven by the uptake and intracellular generation of solutes, which decrease guard cell water potential and create a driving force for water uptake into guard cells. In contrast, during stomatal closure, a reduction of solute contents regulated by membrane transport systems in the guard cells leads to cell deflation and a narrowing of the stomatal aperture [27]. Stomata have evolved an abscisic acid (ABA)-dependent network from the last common ancestor of mosses and vascular plants for drought response [28,29]. The ABA signal transduction system consists of PYR/PYL/RCAR-type ABA receptors, group A 2C-type protein phosphatases (PP2C), and SNF1-related protein kinase 2 (SnRK2) family of proteins, which are key negative regulators of ABA signalling [30]. Once bound to ABA, the receptor complex inactivates PP2C, thereby activating protein kinase SnRK2 [31], which induces the production of ROS [32] and nitric oxide (NO) [33]. Hydrogen peroxide (H_2_O_2_) can activate Ca^2+^ channels in the plasma membrane (PM) of *Arabidopsis* guard cells and inhibit inward K^+^ channels [34,35]. Nitric oxide has also been identified to regulate K^+^ and Cl^−^ channels through a subset of ABA-evoked signalling pathways in guard cells [33]. Guard cell anion channels are activated by Ca^2+^ and become permeable, allowing the efflux of anions [36], and K^+^ outwardly rectifying channels (GORK) are activated by K^+^ loss [37], leading to stomatal closure.

Despite the advancements in understanding retrograde signals and plant membrane transport [22,27], the interactions between retrograde signalling pathways and ion transport across the plasma membrane, tonoplast, and chloroplast membranes are poorly understood. Chloroplasts are also involved in other signalling networks such as sulphate metabolism and signalling. For instance, sulphate transporters play important roles in plant drought and salinity tolerance [38]. Accumulation of sulphate in the leaves enhances ABA biosynthesis in the leaves, ABA sensitivity causing stomatal closure [39]. Newly synthesised ABA is then transported to the roots for signal transduction and the expression of drought stress-responsive genes [40]. Therefore, sulphate and its transporters are suggested as important components in long-distance signalling under drought [38,39]. A compound associated with sulphate metabolism, 3′-phosphoadnenosine 5′-phosphate (PAP), is thought to act as a typical retrograde signal. PAP is produced in chloroplasts under drought stress to induce the expression of nuclear-encoded stress response genes, leading to stomatal closure [25,26]. Whether the PAP signal joins the ABA signalling pathway or whether PAP is part of a separate pathway from ABA is still unclear. In this review, we summarise the progress towards a better understanding of the relationships between retrograde signals and ion transport in adverse environments. We illustrate the possible roles for retrograde signalling in plant stress response using the recently discovered PAP signalling pathway. We also propose a potential interaction whereby chloroplasts sense drought and produce signals (e.g., PAP) which regulate stomatal movement to maintain water potential in plant cells and to guarantee a stable photosynthesis rate under drought.

## 2. Typical Retrograde Signals in Chloroplasts

Retrograde signalling refers to communications from organelles to the nucleus. Chloroplasts, whose primary function is photosynthesis, are also critical for other aspects of plant development and physiology, including the synthesis of amino acids, nucleotides, fatty acids, phytohormones, and the assimilation of sulphur [16]. Several chloroplastic secondary metabolites also function as retrograde signals that are responsible for plant defence against pathogens and for plant adaptation to heat, drought, and high light [20]. Some abiotic stress responses in plants are likely to share common signalling mechanisms or components [41]. For example, 69% of drought-induced genes are also induced by high-light stress, suggesting a strong interconnection between the responses to these two types of stress [42]. This makes abiotic stress defence systems more efficient in plants because genes induced by one type of stress would be efficient also in response to other stress signals. Tremendous progress has been made in identifying retrograde signalling components and their corresponding pathways in chloroplasts. Retrograde molecules include carotenoid oxidation products [43], ROS such as H_2_O_2_ [44], tetrapyrroles [45], phosphoadenosines [25], carbohydrate metabolites [46,47], and isoprenoid precursor methylerythritol cyclodiphosphate (MEcPP) [23]. Retrograde signals include metabolite by-products [48], transcription factors [49], and thylakoid redox state [50] (Table 1).

### 2.1. Reactive Oxygen Species

Plastid ROS molecules can regulate specific proteins and plastid redox-associated nuclear genes (PRANGs) [16] and usually cause oxidation of biomolecules and act as signal molecules in plants [43]. ROS occur as singlet oxygen (^1^O_2_), superoxide anions (O_2_^−^), H_2_O_2_, or hydroxyl radicals (OH^−^), and excessive ROS can lead to programmed cell death (PCD). Plants have developed efficient and versatile scavenging systems to keep ROS under control. Compared with other ROS, H_2_O_2_ appears most likely to be a retrograde signal, because of its small size, lower toxicity, longer half-life, relatively high concentration in cells, and ability to cross cell membranes and move between cell compartments [54].

During plant evolution, ROS have developed many functions associated with plant development and stress tolerance. For instance, H_2_O_2_ is involved in ABA-induced stomatal closure by regulating Ca^2+^, K^+^, and Cl^−^ channels [36,55]. H_2_O_2_ regulates a mitogen-activated protein kinase-like enzyme (MAPK) in *Arabidopsis thaliana* [44], indicating a role for H_2_O_2_ in retrograde signalling. The *MAPK* gene family has been found to co-operate with ABA in plant abiotic stress responses [56]. Experimental evidence suggests a role for ^1^O_2_ in addition to H_2_O_2_ in retrograde signalling. The transient nature of ^1^O_2_and its localised production suggest that it may act via more stable second messengers [57]. Accumulation of ^1^O_2_ occurred in the *fluorescent* (*flu*) mutant of *Arabidopsis* during a shift from dark to light, resulting the differential expression of 70 nuclear genes [57]. FLU is a negative regulator of tetrapyrrole metabolism which over-accumulates the photosensitiser protochlorophyllide in the dark and consequently generates ^1^O_2_ under light [57]. Over-accumulation of singlet oxygen leads to PCD in *flu* leaves but does not occur in double-mutant *ex1 flu* (*execute 1*) [58]. Interestingly, ^1^O_2_ still over-accumulates in *ex1 flu* but stops PCD in leaves, indicating that ^1^O_2_ and EX1 have roles in a retrograde pathway that regulate programmed cell death. 

### 2.2. Tetrapyrrole and Mg-Protoporphyrin

With different structures, along with a variety of ring substituents, tetrapyrroles show different functions [59]. In *Arabidopsis*, the role of tetrapyrroles in retrograde signalling was first identified in studies of genomes uncoupled (*gun*) mutants [60]. Tetrapyrrole-controlled gene expression is evolutionarily conserved in many plant species [51]. GUN1 is located in chloroplasts and functions to impair plastid gene expression [60]. Mg-protoporphyrin (Mg-ProtoIX) acts between the chloroplast and the nucleus in the tetrapyrrole signalling pathway [45]. Accumulation of Mg-ProtoIX regulates the expression of many nuclear genes encoding photosynthesis-associated chloroplast proteins, such as heat shock protein HSP81-2 [45]. The heat shock protein gene *HSP70* can be induced by either exogenous hemin (an oxidized form of heme that is reduced to heme in vivo) and Mg-ProtoIX treatment or light incubation, suggesting that these chemicals may converge in the same pathway [61]. Further evidence shows that heme is produced by plastid ferrochelatase 1 (FC1) in chloroplasts from which heme is exported to regulate gene expression of photosynthesis-related nuclear genes (PhANGs) [48,61]. 

### 2.3. Transcription Factors

Transcription factors also have roles in retrograde signalling. The APETALA 2/ethylene-responsive element binding protein (AP2/EREBP) family of transcription factors has been implicated in hormone, sugar, and redox signalling in relation to abiotic stresses [62]. Members of the Ethylene-Responsive Factor (ERF) subfamily, the largest group of transcription factors among the AP2/EREBP family, were first identified through their regulation of ethylene responses [63]. Among the ERF group, the AP2-like transcription factor Abscisic Acid Insensitive 4 (ABI4) functions in three retrograde signalling processes [64]: tetapyrrole synthesis [45], plastid gene expression (PGE) [65], and photosynthesis electron transfer chain (PET), affecting both photosynthesis- and stress-related genes [65,66]. The pathways associated with ABI4 also involve PHD-type transcription factor with transmembrane domains (PTM), which is located in the chloroplast envelope. In response to retrograde signals, the accumulated mature form of PTM modifies histones, thereby regulating ABI4 transcription [53,67]. Moreover, AP2/EREBP family has also been implicated in retrograde signalling by interacting with redox signalling in abiotic stresses such as cold and drought [49]. A typical example is plastid redox-insensitive 2 (PRIN2), which is a chloroplast component located in the nucleus and which functions in redox-mediated retrograde signalling, specifically by interacting with plastid-encoded RNA polymerase (PEP). PEP functions as a retrograde signal synchronizing nuclear and plastid genomes for the expression of photosynthesis-associated nuclear genes (PhANGs) [51].

WHIRLY (WHY) proteins are activators of nuclear gene transcription [68] and are required for plastid genome stability [69]. WHY1 proteins with the same molecular weight have been found in both chloroplasts and nucleus of the same cell [70]. This suggests that the mature forms of these proteins are intracellularly mobile [52]. Salicylic acid (SA) is a phenolic compound that is involved in plant responses to stresses [71]. WHY1 in *Arabidopsis* participates in both SA-dependent disease resistance and SA-induced expression of systemic acquired resistance (SAR) responsive genes [72], which suggests that WHY1 may act as a retrograde signal. It was proposed that WHY1 may be sensitive to the redox state of the chloroplast, which may cause changes in the polymerisation of WHY1 allowing monomers to translocate to the nucleus to trigger NGE [73].

### 2.4. 3′-Phosphoadenosine 5′-Phosphate (PAP)

PAP is produced in secondary sulphur assimilation as a by-product of the transfer of sulphate from 3′-phosphoadenosine 5′-phosphosulfate (PAPS) to acceptor molecules in a reaction catalysed by sulphotransferases (SOTs) [74]. It was reported that there is a 20-fold increase of PAP levels in *Arabidopsis* under drought stress, and a PAP-accumulating mutant, *altered expression of APX2* (*alx8*) shows considerably improved drought tolerance [75]. Contrasting results were shown concerning the location of the phosphatase SAL1, which functions in dephosphorylating PAP to AMP (Adenosine Monophosphate) to reduce PAP concentrations [25]. Cellular SAL1 has been localized to the chloroplast [76], cytosol [77], and nucleus [78]; however, detailed and more convincing data has confirmed that SAL1 accumulates in the mitochondria and chloroplasts and that PAP is present in chloroplasts [25], which suggests that PAP is transported to the cytosol by a thylakoid ADP/ATP (Adenosine diphosphate/Adenosine triphosphate) carrier such as PAPST1 [79]. Once in the nucleus, PAP regulates drought stress-responsive gene expression (such as the expression of *APX2*, *ZAT10*, *DREB2A*) [25]. All this evidence suggests there is a SAL1–PAP retrograde pathway that alters NGE during drought stress.

### 2.5. Methylerythritol Cyclodiphosphate (MEcPP)

In plants, isoprenoids such as MEcPP are synthesised via two different pathways, i.e., the cytosolic mevalonate pathway and the 2-C-methyl-d-erythritol 4-phosphate pathway [80], and regulate a specific set of stress-responsive nuclear-encoded plastidial proteins [23]. *Hydroperoxide lyase* (*HPL*) is a stress-inducible nuclear gene encoding a plastid-localised protein in the oxylipin pathway that produces compounds for plants’ response to biotic and abiotic stresses [81]. SA has broad roles in regulating important plant physiological processes such as photosynthesis [82], antioxidant defence [83], and water maintenance under drought stress [71]. MEcPP causes high levels of SA and HPL, so that plants under stress have higher levels of MEcPP. Thus, MEcPP acts as a retrograde signalling molecule that induces expression of stress-related genes [23].

## 3. Linking Retrograde Signals to Chloroplastic Ion Transporters under Stress

Since chloroplast-initiated retrograde signals are involved in transducing various environmental stresses, and chloroplastic ion transporters are significant in regulating chloroplast status [84], do chloroplast ion transporters affect the generation of retrograde signals? Three general categories of proteins have been classified as ions transporters, i.e., channels/porins, primary transporters/pumps, and secondary transporters [85]; these have been shown to have important roles in photosynthesis. Here, we focus on the chloroplastic ion transporters that are involved in retrograde signalling. For details of channels or transporters related to photosynthesis, readers are directed to two excellent reviews [84,86].

### 3.1. Ion Transport, Retrograde Signalling and ROS-Regulated Photosynthesis in Chloroplasts

Chloroplastic ion transporters may participate in retrograde signalling via the synthesis of retrograde signals or of intermediates such as ROS and chloroplasts are the source and target of cellular redox regulation [87]. Many chloroplastic transporters affect chloroplast redox status and, therefore, determine the photosynthetic rate. ROS production from chloroplasts has been regarded as a key retrograde signal, affecting NGE [16,88,89]. It was suggested that accumulation of H_2_O_2_ specifically in the chloroplasts induces the expression of nuclear-encoded genes, such as cytoplasmic *ascorbate peroxidase 2* (*APX2*) [90]. Silencing a thylakoid membrane-bound APX (*tAPX*) gene regulates H_2_O_2_, resulting in increased levels of oxidized proteins in chloroplasts. However, the expression of ROS-responsive genes was negatively regulated in a *tAPX* silenced mutant, which suggests H_2_O_2_ triggered retrograde signalling from chloroplasts under stress [91]. Indeed, photosynthetic redox state and ROS production have been proposed to be modulated by the status of plastoquinol (PQH_2_) and plastoquinone (PQ) in chloroplasts [92] as well as by chloroplastic ion transporters [84]. 

**Figure 1:**
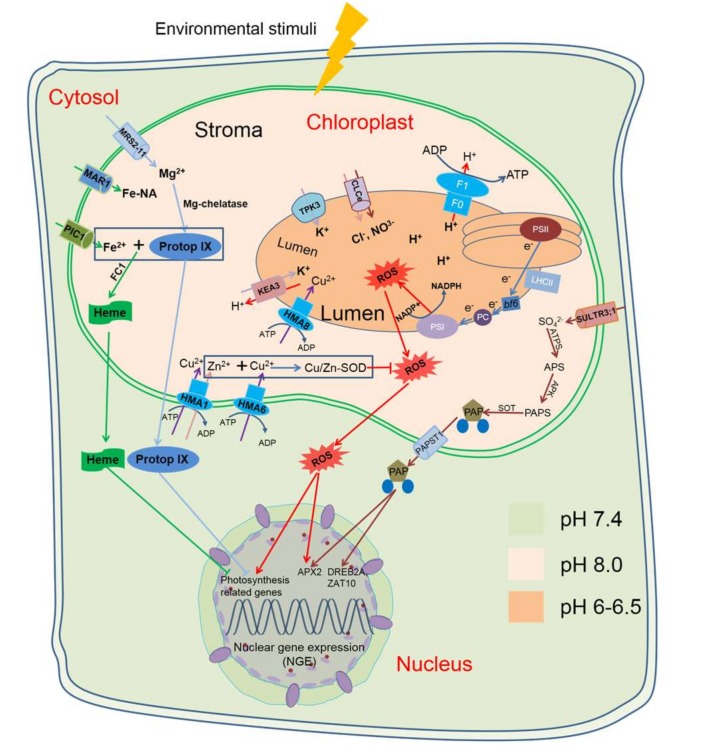
A schematic diagram of typical retrograde signalling pathways in plant cells. High-light stress could induce ^1^O_2_ accumulation which causes the accumulation of β-cyclocitral in the chloroplast. β-cyclocitral is exported to the nucleus to regulate expression of defense genes [43]. Elements in the tetrapyrrole pathways act as retrograde signals. Mg-ProtoIX and heme can both be regulated by FC1 and then transported from the chloroplasts to the nucleus to regulate photosynthesis-related genes [48,61]. Methylerythritol 4-phosphate (MEP) pathways also participate in retrograde signalling pathways, and high light could also induce methylerythritol cyclodiphosphate (MEcPP) production in chloroplasts and then regulate nuclear *HPL* gene expression [23]. PAP (3′-phosphoadnenosine 5′-phosphate), induced by drought and high light, could be transferred from the chloroplasts to the nucleus and regulate the expression of a set of genes [25]. Abbreviations: Protop IX, Protoporphyrin IX; FC1, ferrochelatase 1; TPK3, Tandem-pore K^+^ selective channel3; KEA 3, Cation/proton antiporter 3; CLC, anion channel of Cl^−^ channel (CLC) family; ROS, reactive oxygen species; PSI and PSII, Reaction centres of photosystem I and II; HMA, P-type ATPase of *Arabidopsis*/Heavy-metal-associated; bf6, cytochrome b6f complex; PC, plastocyanin; LHCII, Light harvesting complex; SULTR, phloem-localized sulphate transporter; ATPs, ATP sulphurylase; APS, Adenosine 5′-phosphosulfate; APK, APS kinase; PAPS, 3′phosphoadnosine 5′-phosphosulfate; SOT, Sulfotransferase; PAP, 3′-Phosphoadnenosine 5′phosphate; APX, Ascorbate peroxidase 2; DREB2A, Drought responsive element binding 2A; ZAT10, Salt tolerance Zinc Finger.

The natural light environment of plants changes rapidly and has driven to the evolution of sensing mechanisms that allow the efficient acclimation of plants to light conditions [93]. In chloroplasts, thylakoids are packed to occupy a small volume with a large surface area, and thylakoid membranes also have the highest known protein-to-lipid ratio among all membranes. They contain six major protein complexes, namely, light-harvesting complexes I (LHCI) and II (LHCII), photosystems I (PSI) and II (PSII), Cytochrome b6f, and ATP synthase CF0F1 [94]. Electron transport should be balanced among these two photosystems (PSI and PSII) for NADPH and ATP production; an imbalance in electron flow can occur during stress conditions. This imbalance can lead to excess electron excitation, photoinhibition, and elevation of ROS. Under high light, induced adaptive structural changes, such as the swelling of thylakoids and an increase in the partition gaps between the thylakoids, can occur [95]. Therefore, the chloroplastic ion channels and transporters located in thylakoids become important, as these transporters are responsible for preventing membrane depolarization or hyperpolarization due to the excessive accumulation of cations and anions via ion transporters [96]. Light energizes H^+^-ATPase in thylakoid membrane to generate the H^+^-motive force necessary for ion and solute transport during photosynthesis [97]. However, the H^+^ concentration in the different compartments of chloroplasts needs to be well regulated; for example, stromal pH is maintained at pH 8.0 while the luminal pH is around pH 5.5–6.2 [98]. The luminal acidification is necessary to activate non-photochemical quenching (NPQ), which alters LHCII and allows part of the excitation energy to be dissipated to prevent ROS production [84]. All these processes are likely to require ion and solute homeostasis regulated by ion channels, pumps, and co-transporters to maintain the balance between chloroplasts and the rest of the cell (Figure 1).

### 3.2. Direct Modulation of Chloroplastic Retrograde Signalling by Ion Transporters

Some ion transporters have roles in regulating ion homeostasis and balancing ROS production, but their direct interaction with retrograde signals is still elusive. In *Arabidopsis*, a thylakoid membrane two-pore potassium channel, i.e., TPK3, was shown to regulate H^+^ concentration through ion counterbalancing [99]. TPK3-silenced plants display impaired CO_2_ assimilation and reduced non-photochemical dissipation of excessively absorbed light [99]. The K^+^/H^+^ and antiporter KEA3 is responsible for the recuperation of luminal K^+^ concentration during the night [100] and for optimizing photosynthesis [101]. A member of the Cl^−^ channel (CLCs) family, CLCe, has been identified in thylakoid membranes for ion counterbalancing in chloroplasts during light-driven proton transfer across the thylakoid [102]. However, under environmental stresses such as drought, the balance is disrupted, resulting in high ROS production in chloroplasts. Photosynthetic electron transport-generated redox signals in chloroplast also control PhANGs [103] (Figure 1). 

### 3.3. Indirect Regulation of Chloroplastic Retrograde Signalling by Ion Transporters

Other chloroplast-located ion transporters responsible for transporting specific ions for the biosynthesis of retrograde signals have been identified by proteomic studies [104]. Since Cu is critical for electron transport and ROS scavenging in chloroplasts, undoubtedly, chloroplast-located Cu transporters play critical roles in reducing ROS production. Copper in chloroplasts has two forms: reduced and oxidized. Cu^2+^ is the redox cofactor of plastocyanin (PC), the protein required for transferring electrons from the cytochrome b6f complex to PSI [105], leading to lower ROS production in chloroplasts. Cu is also one of the components of Cu/Zn superoxide dismutase (Cu/Zn-SOD), which functions in scavenging ROS produced during photosynthesis under stress conditions [100]. In *Arabidopsis*, three proteins (HMA1, HMA6, and HMA8) are involved in Cu homeostasis. HMA1 and HMA6 are located in the chloroplast envelope and are involved in importing Cu into the chloroplast for Cu/Zn-SOD synthesis [106,107], while HMA8 is found in the non-appressed fractions of thylakoid membrane required for PC biosynthesis [108] (Figure 1). The *Arabidopsis* mutant *hma1* has lower chloroplast copper content and a diminution of the total chloroplast SOD activity, which is essential for ROS reduction under stress [106].

Being a component involved in all photosystems and an important redox-active metal ion critical for photosynthetic electron flow, iron is involved in various chelation and oxidation/reduction steps that affect ROS production [109]. However, Fe homeostasis must be fine-tuned because excessive free Fe promotes the formation of free radicals via the Fenton reaction in plants. Ferritins are ion-storage proteins, responsible for either sequestering or releasing iron upon demand [110]. Transgenic plants overexpressing the wheat ferritin gene *TaFER-5B* exhibited enhanced temperature, drought, oxidative, and iron stress tolerance associated with ROS scavenging [111]. Most importantly, chloroplastic Fe transporters may participate in retrograde signalling by importing Fe into chloroplasts for the biosynthesis of heme, a key retrograde signaling molecule [48,61] (Table 1 and Figure 1). Biosynthesis of heme needs ferrochelatse I (FC I), which catalyses the insertion of Fe^2+^ into protoporphyrin IX (ProtoP IX) to form heme [112]. The *Arabidopsis* Permease Chloroplasts 1 (PIC1), which contains four predicted α-helices targeted to the inner envelope, is involved in iron transport in chloroplast [113]. Moreover, Multiple Antibiotic Resistance 1 (MAR1), which is a homolog of the ferroportin efflux transporters, was also identified as mediator of the transport of Fe or Fe-chelating polyamines such as nicotianamine into chloroplasts [114].

Magnesium (Mg) is another element essential for heme retrograde signalling via Mg^2+^ insertion into ProtoP IX by Mg-chelatase from Mg-protoporphyrin, the precursor of chlorophyll and heme biosynthesis [61]. In *Arabidopsis*, a putative Mg^2+^ transporter, MRS2-11, is located in the chloroplast envelop and is responsible for Mg transport [115]. Mg-protoporphyrin can also be regarded as a retrograde signaling molecule, regulating the expression of PhANGs [116]. Moreover, sulphate transporters are also likely to participate in the biosynthesis of retrograde signals by importing ions into chloroplasts. The sulphate transporter SULTR3;1 is located in the chloroplast membrane and is responsible for sulphate uptake into chloroplasts [117]. For instance, another retrograde signal PAP is synthesised from sulphate [74,118] and is capable of moving between chloroplasts and cytosol to upregulate *APX2* and *drought-responsive element binding protein 2A* (*DREB2A*) under high-light and drought stress [25,41,75].

In summary, chloroplastic ion transporters are coordinated to regulate ion fluxes and electron transport between all the photosystems in chloroplasts. These transporters may also participate in retrograde signalling pathways either through affecting ROS production or through delivering the components required for the synthesis and regulation of the retrograde molecules. As a balanced electron flux needs to be maintained in chloroplasts, we propose that chloroplast-derived retrograde signals are potential feedback signals that regulate chloroplastic ion transporters. However, few studies have addressed whether retrograde signals like ROS regulate ion transporters in chloroplast membranes. Patch clamp measurements on chloroplasts and membrane patches will be essential to explore the interactions between retrograde signals and membrane transport.

## 4. Linking Retrograde Signals to Ion Transport for Stomatal Regulation

**Figure 2:**
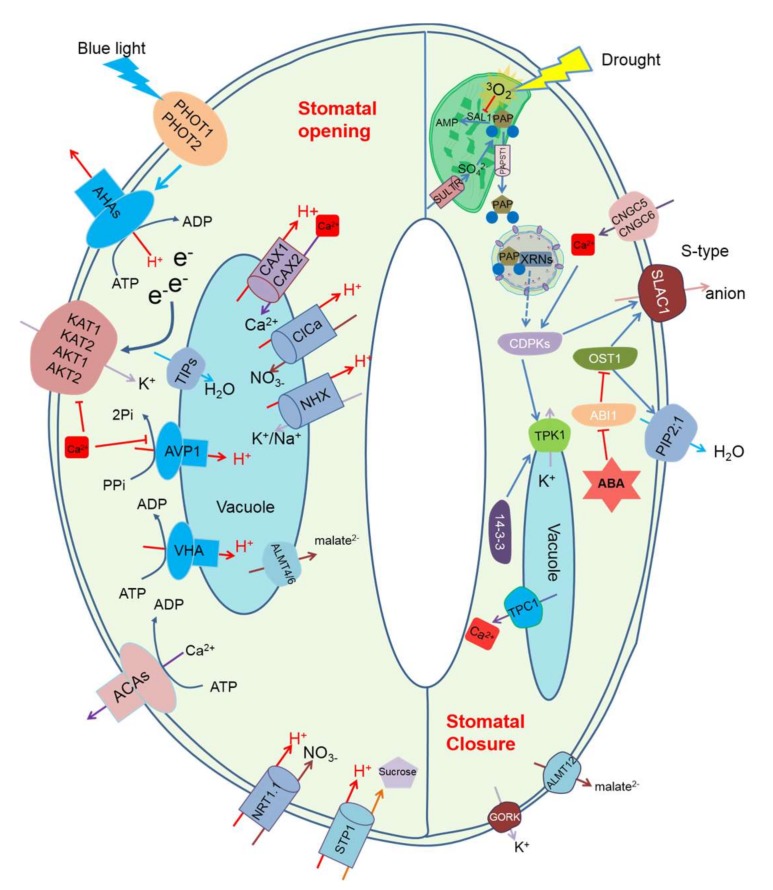
A schematic diagram of chloroplast-located ion transporters and retrograde signal molecule 3′-phosphoadnenosine 5′-phosphate (PAP), and their roles in stomatal regulation. PHOT1 and PHOT2 sense blue light, which activates plasma membrane proton pump AHAs, and this leads to the efflux of H^+^ from cytosol [119]. The accumulated electrons on the cytosolic side lead to activation of plasma membrane-located potassium inward-rectifying channels [120], leading to K^+^ influx. However, these potassium inward-rectifying channels can be inhibited by cytosolic Ca^2+^ accumulation [121]. CNGCs and CAXs are responsible for cytosolic Ca^2+^ accumulation [36], and CAX can be inactivated by ABA, which increases cytosolic Ca^2+^ accumulation [122]. ABA also inhibits blue light-induced H^+^-ATPase activation, which leads to stomatal closure [123]. Sulphate can be transported into chloroplasts by SULTR for the biosynthesis of PAP [117,118]. PAP is degraded by SAL1/ALX8 to AMP [25]. Under drought stress, ROS production in chloroplasts reduces SAL1 activity, which leads to PAP accumulation in the protoplast [25]. PAP is then transported into the cytosol by PAP transporter, PAPST1 [79], from where it moves to the nucleus to bind to the stress response genes XRNs, which potentially leads to CDPKs expression [26]. CDPKs activate SLAC1 channels, which leads to anion efflux [26]. Cytosolic Ca^2+^ also has a role in regulating CDPKs [124]. Besides, CDPKs and protein 14-3-3 have a role in regulating vacuole potassium channels activity [125,126]. ABA-induced stomatal closure depends on OST1 activity. OST1 has a role in activating anion efflux and inhibits water aquaporin channel PIP2;1 activity [127,128], which leads to stomatal closure. Abbreviations: PHOT, phototropins; AHA, Plasma membrane H^+^-ATPase; ATP, adenosine triphosphate; ADP, Adenosine diphosphate; KAT1, K^+^ channel 1 in *Arabidopsis*; KAT2, K^+^ channel in *Arabidopsis* 2; AKT, *Arabidopsis* Thaliana Rectifying channel ; ACA, Ca^2+^-ATPase; CNGC, *Arabidopsis* Cyclic nucleotide-gated ion channels; NRT1.1, Nitrate Transporter 1.1; STP1, Sugar Transporter 1; ABA, Abscisic acid; ALMT, Aluminium-activated malate transporter; VHA, vacuolar H^+^-ATPase; AVP, vacuolar H^+^/K^+^-PPase; TIPs, Tonoplast Intrinsic Proteins; CAX, Cation Exchanger; CLCa, Chloride Channel a; NHX, Na^+^,K^+^/H^+^ antiporters; AMP, Adenosine Monophosphate; SAL1, Altered expression of APX2; PAP, 3′-phosphoadnenosine 5′-phosphate; SULTR, phloem-localized sulphate transporter; PAPST1, 3′-Phosphoadenosine 5′-Phosphosulfate Transporter 1;ABI, ABA Insensitive; OST1, Open Stomata 1; TPC, Two-pore Ca^2+^ channel; TPK, Two-pore K^+^ channel; CDPKs, Ca^2+^ dependent protein kinases; SLAC1, Slow Anion channel-associated 1; PIP2;1, Plasma Membrane Intrinsic Protein 2; GORK, Guard Cell Outwardly Rectifying K^+^ channel.

The retrograde signal PAP regulates stomatal closure, enhancing drought tolerance [25,26]. In addition, drought tolerance is partially controlled by the activity of pumps, ion channels, and cotransporters located in the plasma membrane and tonoplast of guard cells, which generate ion gradients, regulating stomatal opening and closure [6,12] (Figure 2). Are there any links between retrograde signals and these membrane transporters? Published reports showing direct links between retrograde signals and ion transporters at the plasma membrane and tonoplast are still limited. Here, we focus on a key component of drought tolerance, i.e., membrane transporters that regulate stomatal opening and closure. We illustrate the potential interactions between retrograde signals and ion transporters in the context of stomatal guard cells and drought tolerance.

## 5. Plasma Membrane Transport in Stomatal Guard Cells

### 5.1. Plasma Membrane Pumps 

The plasma membrane H^+^-ATPase (AHA) protein family has many members in different plant species [6], and these proteins are responsible for H^+^ movement by coupling with ATP hydrolysis, which is the primary motive force for stomatal movement [129]. In guard cells, blue light, ABA, auxin, and exogenous Ca^2+^ play roles in H^+^-ATPase regulation [130,131,132]. For instance, the blue light receptor phototropins (PHOT1 and PHOT2) [133] sense blue light and activate plasma membrane H^+^-ATPases, which results in an efflux of H^+^ from the cytosol [119]. The activated H^+^-ATPase induces hyperpolarization which in turn induces K^+^ uptake via inward-rectifying K^+^ channels [120]. Conversely, ABA strongly inhibits blue light-induced H^+^-ATPases activation, which leads to stomatal closure. ABA-induced Ca^2+^ accumulation in the cytosol also inhibits H^+^ pumping and ATP hydrolysis in guard cells [123]. For example, Mg-chelatase H subunit (CHLH), which was found to mediate chlorophyll biosynthesis, regulates stomatal closure in part through dephosphorylating and inhibiting guard cell H^+^-ATPase [134]. Ca^2+^-ATPases (ACAs) regulate Ca^2+^ homeostasis on different membranes in a range of plant cell types in response to stress [135,136,137,138]. Ca^2+^-ATPases ACA8 and ACA10 were found to be targeted to guard cells in *Arabidopsis*. The expression of *ACA8* was found to be upregulated, while that of *ACA10* was downregulated by cold treatment, and the promoter of *ACA8* has been shown to contain cold-responsive C-repeat/dehydration-responsive element motif [136]. BONZAI1 (BON1) interacts with the autoinhibitory domains of ACA10, ACA8, and ACA10/8, and it functions in the generation of cytosol calcium signatures that are critical for stomatal movement [137].

### 5.2. Plasma Membrane Ion Channels

Potassium (K) participates in plant growth and development [139] and also affects the homeostasis of other processes [140]. Potassium channels located in guard cell plasma membranes play a critical role in K^+^ uptake and release, thus modulating guard cell turgor and volume [141]. In *Arabidopsis*, inward K^+^ channels (e.g., KAT1, KAT2, AKT1, AKT2) and outward K^+^ channels (e.g., GORK) were identified as responsible for the K^+^ fluxes during stomatal movement [142]. For example, the *Arabidopsis* mutant *gork*, that has a non-functional gated outward-rectifying K^+^ channel, showed impaired stomatal closure [37]. In addition, overexpressing *KAT1* impaired stomatal movement in *Arabidopsis* [143], but stomatal behaviour of a knockout mutant *kat1* was little affected [144]. Guard cell K^+^ channels can be regulated by ABA, cytosolic Ca^2+^, pH, protein kinases, and phosphatases [121,142,145]. All these results suggest that potassium channels are downstream in guard cell signalling pathways and directly affect stomatal movement by changing cell turgor. 

Ca^2+^ accumulation in the cytosol is one of the most important processes in ABA-induced stomatal closure, and many voltage-dependent Ca^2+^-permeable channels were identified in the plasma membrane of different types of plant cells [6,146]. Cyclic nucleotide-gated ion channels (CNGC)-mediated cytosolic Ca^2+^ rise contributes to the dynamic regulation of guard cell anion channels and stomatal closure [36,147]. *Arabidopsis* CNGC5 and CNGC6 have been identified as plasma membrane Ca^2+^ channels that are highly expressed in guard cells. Guard cells in the *cngc5cngc6* double mutant exhibited dramatically impaired cGMP-activated currents. Moreover, the guard cells of the double mutant exhibited functional ABA-activated hyperpolarization-dependent Ca^2+^-permeable cation channel currents, intact ABA-induced stomatal closing responses, and whole-plant stomatal conductance responses to darkness and changes in CO_2_ concentration [148]. It has been reported that Ca^2+^ functions downstream of ROS, and, for instance, a Ca^2+^ current is activated by ROS in ABA-induced stomatal closure [149].

There are two major types of anion channels located in the plasma membrane: rapid (R-type) and slow (S-type) anion channels [6,147,150]. An anion efflux is essential for stomatal closure through both R-type [151] and S-type [152,153] anion channels. Both types of anion channels can be activated by cytosolic Ca^2+^ and are permeable to a range of anions, including Cl^−^, malate^2−^, and NO^3−^ [27]. Plasma membrane *Aluminium-activated malate transporter* (*ALMT12*) is highly expressed in *Arabidopsis* guard cells, and plants lacking ALMT12 are impaired in ABA-induced stomatal closure. ALMT12 is capable of transiently depolarising guard cells to trigger membrane potential oscillations and initiates long-term anion and K^+^ efflux via slow anion channel 1 (SLAC1) and GORK, respectively [151].

Water channels, or aquaporins, have varied functions in stomatal regulation and transport of H_2_O, CO_2_, and H_2_O_2_ [154]. For instance, knocking out the plasma membrane Intrinsic Protein (PIP) PIP2;1 in *Arabidopsis* leads to a defect in ABA-induced stomatal closure in *pip2;1* plants. In addition, work using *Xenopus laevis* oocytes has shown that PIP2;1 water transport activity is increased when open stomata 1 (OST1) phosphorylates a cytosolic PIP2;1 peptide at Ser-121. ABA-triggered stomatal closure requires an increase in guard cell permeability to water and possibly H_2_O_2_, through OST1-dependent phosphorylation of PIP2;1 [128].

### 5.3. Plasma Membrane Cotransporters

Many plasma membrane cotransporters regulate stomatal movement and play a role in stress tolerance [6,155]. Here, we only present three key examples of cotransporters. The dual-affinity nitrate transporter gene, *NRT1.1*/*CHL1*, is expressed in *Arabidopsis* guard cells, and the *chl1* mutant shows enhanced drought tolerance. It was reported that *chl1* mutants showed reduced nitrate accumulation in guard cells during stomatal opening and failed to show nitrate-induced depolarization of guard cells [156]. The guard cells of several plant species were shown to accumulate sucrose as an osmoticum that drives water influx to increase stomatal aperture, and an *Arabidopsis* H^+^-monosaccharide symporter, STP1, was identified in guard cells. A transient increase in *STP1* expression correlates in time with the described guard cell-specific accumulation of sucrose, and a role for STP1 in monosaccharide import into guard cells has been reported [157]. Moreover, the ATP-binding cassette (ABC) transporter gene *MRP4* is highly expressed in stomata and MRP4 is localized to the plasma membrane in *Arabidopsis*. Stomatal aperture in three independent *mrp4* mutant was larger than in wild-type plants, indicating the involvement of MRP4 in the complex regulation of stomatal aperture [158].

### 5.4. Tonoplast Transport in Stomatal Guard Cells

#### 5.4.1. Tonoplast Pumps

Vacuolar acidification requires the combined activity of vacuolar type H^+^-ATPase (V-ATPase) and tonoplast inorganic pyrophosphatase (V-PPase), both of which are key determinants for stomatal regulation and stress response [147,159,160]. V-ATPase maintains a proton electrochemical gradient across endomembrane compartments, including the vacuole [147,161]. V-ATPase is highly abundant, representing 6.5–35% of the total tonoplast proteins in different species [162]. This enzyme is composed of several polypeptide subunits that are located in two major domains, a membrane peripheral domain (V_1_) and a membrane integral domain (V_0_) [162]. The expression of all V-ATPase subunits can be increased in response to salt stress [162], and expressing *Arabidopsis VHA-C* in *Hordeum vulgare* improved plant performance under saline conditions [163]. Further evidence showed that ABA significantly increases V-ATPase H^+^-transport activity [164]. This suggests an important role of V-ATPase in regulating plant stress tolerance. V-PPase activity in guard cells is involved in stomatal regulation [165]. Guard cell protoplasts of *Vicia faba* exhibited hydrolytic activity characteristic of tonoplast-localized V-PPase. The activity was inhibited by a specific V-PPase inhibitor and by cytosolic Ca^2+^ and stimulated by K^+^. V-PPase AVP1 controls auxin transport and, consequently, auxin-dependent development [159,166]. Expression of an *Arabidopsis AVP1* in cotton improves drought and salt tolerance [167]; however, the role of Ca^2+^-ATPases in stomatal regulation is still elusive. 

#### 5.4.2. Tonoplast Ion Channels

Stomatal closure requires the release of large amounts of K^+^ from guard cells, mostly from the vacuoles [168]. Therefore, vacuolar K^+^ channels are key components in regulating stomatal closure. The *Arabidopsis* genome contains five genes that encode two-pore K^+^ channels (TPK), and TPK1 is located in vacuolar membranes where it mediates K^+^-selective currents between the cytosol and the vacuolar compartments. TPK1 plays a role in intracellular K^+^ homeostasis, slows stomatal closure kinetics [169], and is activated by 14-3-3 proteins [125] and calcium-dependent protein kinases (CDPKs) [126]. In vacuoles, the *Arabidopsis* two-pore channel 1 gene, *TPC1*, encodes a slow vacuolar channel with high affinity for Ca^2+^ permeation [170]. A *tpc1* knockout mutant was shown to lack functional slow vacuolar channel activity and to be defective in ABA-induced stomatal closure because of a poor Ca^2+^ efflux from guard cell vacuoles, which suggests a critical role for intracellular Ca^2+^-release channels in the physiological processes of plants [171,172]. Aluminum-activated malate transporters (ALMTs) are malate channels involved in vacuolar malate accumulation and in tolerance to aluminum [173,174]. In *Arabidopsis*, ALMT9 is a malate-activated vacuolar chloride channel required for stomatal opening [175], whereas ABA-induced stomatal closure involves the phosphorylation-dependent vacuolar anion channel ALMT4 [176] and the vacuolar malate channel ALMT6 in guard cells, both of which are subject to multiple regulation processes [177]. Moreover, the *Arabidopsis* nitrate transporter CLCa is localized in the tonoplast and is able to accumulate nitrate in the vacuole to regulate stomatal movement [178]. While much effort has substantiated the importance of water channel PIPs, comparably little is known about the function of intracellular aquaporins, such as tonoplast intrinsic proteins (TIPs) [179]. For instance, sunflower SunTIP7 and SunTIP20 are guard cell-localized aquaporins, and their expression in *Xenopus* oocytes caused a marked increase in water permeability. The transcript levels of SunTIP7 were markedly and systematically increased during drought-induced stomatal closure, suggesting that SunTIP7 regulates guard cell volume and stomatal aperture [180].

#### 5.4.3. Tonplast Cotransporters

Many vacuolar cotransporters regulate stomatal movement and play a role in stress tolerance [6,181,182]. Here, we only present a couple of key examples: Na^+^, K^+^/H^+^ antiporters (NHXs) and vacuolar cation exchangers. NHXs are involved in K^+^ homeostasis, pH regulation, and salt tolerance [182]. Tonoplast-localized NHX1 and NHX2 are highly expressed in guard cells, but *nhx1*/*nhx2* mutant plants showed defective stomatal function and had reduced ability to maintain the vacuolar K^+^ pools. Thus, NHX proteins are essential for active K^+^ uptake into the tonoplast, for turgor regulation, and for stomatal function [183,184]. Vacuolar cation exchangers CAX1 and CAX3 are involved in mediating calcium transport from the cytosol to the vacuoles using the proton gradient across the tonoplast [122]. Inhibition of ABA-induced stomatal closure by indole-3-acetic acid (IAA) is impaired in the *cax1*/*cax3* double mutant. The *cax1*/*cax3* mutant exhibited constitutive hyperpolarisation of the plasma membrane with a higher apoplastic pH than the wild-type plant. Lower extracellular pH fully restored IAA inhibition of ABA-induced stomatal closure in the *cax1*/*cax3* mutant [185].

## 6. Retrograde Signals and Ion Transport in Drought-Induced Stomatal Closure

Stomatal guard cell turgor is regulated by cell solute concentration, thus ion channels or transporters in cells determine stomatal movement. Each membrane is equipped with a unique set of ion transporters that enables transport of nutrients, solutes, and metabolites [84]. Furthermore, retrograde signals are regulators that enable plants to survive adverse environments. The substantial knowledge of ion transport in stomatal guard cells and the deep understanding of many retrograde signals summarised above have enabled the dissection of these two types of processes and allowed the identification of their complex interactions (e.g., ABA signalling). Here, we present some emerging evidence of these potential interactions.

In a recent report, chloroplast-derived PAP accumulation induced stomatal closure in *Arabidopsis* [26]. PAP increased K^+^ and Cl^−^ efflux from stomatal guard cells, suggesting a role for potassium and anion channels in PAP-induced stomatal closure [26]. Therefore, potential interactions may occur between a drought-related retrograde signal PAP and plasma membrane-located ion transporters. The SAL1–PAP signalling pathway has been identified as a typical retrograde signal with multiple roles, such as regulating programmed cell death [186], and also functions in drought and high-light signalling [25]. Drought-induced ROS production in chloroplasts inhibits SAL1 activity [187], which leads to PAP accumulation and transport to the nucleus. PAP accumulation could activate downstream signalling through binding to nuclear exoribonucleases (XRNs), transcriptionally up-regulating multiple signalling proteins. These proteins, including four CDPKs, activate SLAC1 anion channel activity for stomatal closure under drought [26] (Figure 2). This discovery opens the door to future research on retrograde signals and membrane transport in plant stress tolerance.

## 7. Concluding Remarks and Future Perspectives

**Figure 3:**
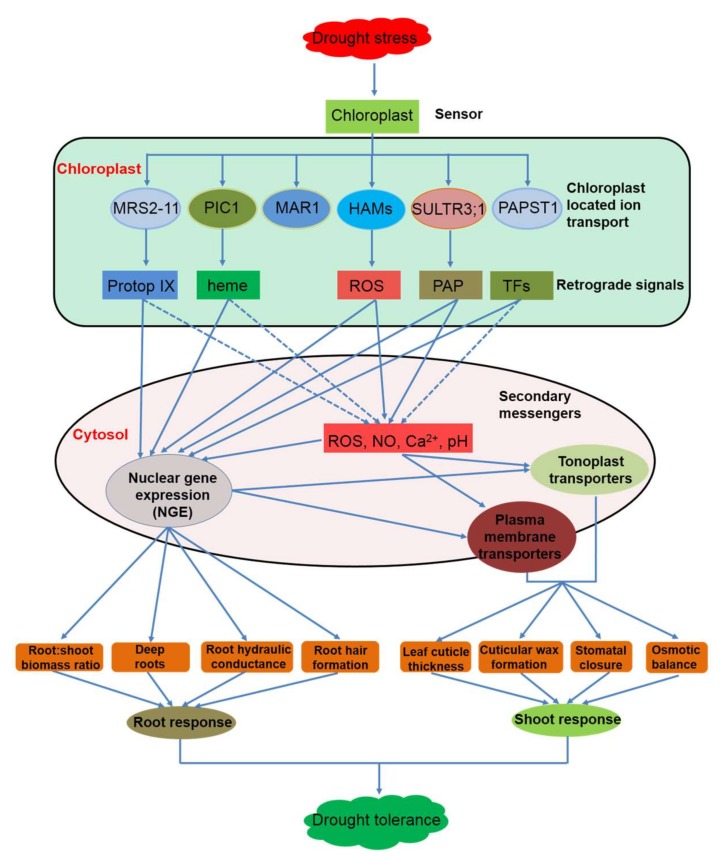
A schematic diagram of retrograde signals and potential mechanisms in regulating plant drought tolerance. Drought can be perceived by chloroplasts. Chloroplast-located ion channels participate in biosynthetic processes of retrograde signals, such as Mg-Protop IX [45], heme [48], ROS [100], and PAP [117], which target either nuclear genes expression (NGE) [48,54,116] or secondary messengers [55]. Secondary messengers and NGE regulate plasma membrane or tonoplast ion transporters [171], triggering the root and shoot responses to drought. Dotted blue arrows: potential interactions. Abbreviations: see legends of Figure 1 and Figure 2.

In this review, we summarised some retrograde signals that participate in the regulation of plant stress tolerance (Figure 1 and Table 1). We compared the chloroplastic transporters that modulate retrograde signalling through retrograde biosynthesis or as critical components in retrograde signalling (Figure 1). We also discussed the roles of important plasma membrane and tonoplast ion transporters that are involved in regulating stomatal movement (Figure 2). Moreover, we illustrated that chloroplast retrograde molecules and plasma membrane- or tonoplast-located ion transporters may interact to regulate plant drought tolerance (Figure 3).

This review highlights some significant questions that need to be addressed. Do chloroplast-initiated retrograde signals and chloroplastic ion transporters regulate each other, and if so, how? How do plant cells establish the interactions between retrograde signals and ion transporters at the plasma membrane and tonoplast? Research is obviously required to identify additional proteins located in the three chloroplast membranes and to study the effects of retrograde signals on transporters in these membranes.

## Figures and Tables

**Table 1:** Source of typical retrograde signals in plants.

Group	Typical Member	References
β-Carotene	β-cyclocitral	[43]
ROS	^1^O_2_	[50]
	H_2_O_2_	[44]
Tetrapyrrole	Mg-ProtoIX	[51]
	heme	[48]
Sulfation	PAP	[25]
Kinases	MAPK6	[47]
Methylerythritol isoprenoid	MEcPP	[23]
Transcription factors	AP2	[49]
	Whirly1	[52]
Chloroplast envelop proteins	PTM/PHD	[53]
